# Reply to the Comment on “Influence of Solvents and Halogenation on ESIPT of Benzimidazole Derivatives for Designing Turn-on Fluorescence Probes”

**DOI:** 10.1021/acsomega.5c04406

**Published:** 2025-05-27

**Authors:** Murillo H. Queiroz, Joel. L. Nascimento, Tiago V. Alves, Roberto Rivelino, Sylvio Canuto

**Affiliations:** † Departamento de Físico-Química, Instituto de Química, 28111 Universidade Federal da Bahia, Rua Barão de Jeremoabo, 147, 40170-115 Salvador, Bahia, Brazil; ‡ Instituto de Física, 28111 Universidade Federal da Bahia, 40210-340 Salvador, Bahia, Brazil; § Instituto de Física, 28133 Universidade de São Paulo, CP 66318, 05315-970 São Paulo, SP, Brazil

Recently, Antonov et al.^1^ have considered a more exhaustive conformational analysis than that done in our work about the influence of solvents and halogenation on ESIPT of benzimidazole derivatives.^2^ They employed a different computational method to investigate the stability of the conformers involved in the process. These authors also argue that our conformational analysis is not adequate, since various additional conformers can be energetically accessible in solution during ESIPT. In fact, at room temperature and a solution concentration of 10^–5^ mol/L, several conformers might statistically contribute to the absorption/emission processes.^3^ However, on average, few conformers really might influence the spectral region where the process occurs.

We clarify here that our selected conformer, referred to as III in ref 2 (see Figure 1a in this Reply), was not arbitrarily chosen but guided by experimental data,^3^ followed by full geometry optimization at the B3LYP/6-31G­(d,p) level, with implicit solvation effects included. The recent calculations performed by Antonov et al.^1^ utilize M06-2X/TZVP, which appears to be useful for describing the tautomerization process of benzimidazole derivatives in the ground state.^1^ Indeed, recent works involving computational chemistry show that the M06-2X functional is sufficiently accurate to describe conformational energy barriers.^4^ However, these new results do not invalidate our main conclusion obtained based on the B3LYP/6-31G­(d,p) calculations.

**Figure 1:**
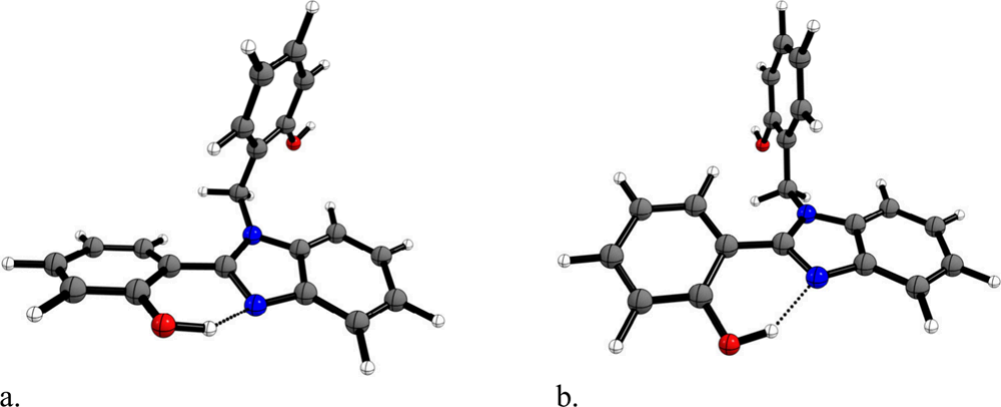
Optimized structures of the nonsubstituted compound with B3LYP/6-31G­(d,p) for conformer III (a), as proposed in ref 2, and conformer III′ (b), representing an alternative geometry of conformer III.

Since several conformers can exist for the different benzimidazole derivatives in their ground-state enolic forms, it is unlikely that all of these possible geometries equally contribute to the photochemical process. Only statistically relevant structures should contribute to the calculated properties.^5,6^ For example, by performing a systematic/stochastic conformational analysis^7^ starting from conformer III, we have obtained about 30 new conformers. To carry out these simulations in a short period of time, we first have employed the HF/3-21G level, as implemented in the Torsiflex program.^8^ Later, by performing a full reoptimization of the structures with B3LYP/6-31G­(d,p) including the implicit solvent effect, i.e., within the same level of theory employed in ref 2, we have obtained 28 conformers. However, only eight of them exhibited lower energies (Table S1). Indeed, we have found a similar enolic tautomer (see Figure 1b) to that found by Antonov et al.^1^ as being the most stable structure in our sampling.

Considering the new conformer (III′), our calculations at the B3LYP/6-31G­(d,p) level yield ΔE_(III–III′)_ = 2.2 kcal/mol. This difference increases to 2.5 kcal/mol when one employs M06-2X/TZVP, as also tested by us. However, the vibrational frequency associated with the interconversion III ↔ III′ is very low (about 19–22 cm^–1^) indicating a small energy barrier between both conformers. Using a guess to find a possible transition state (Figure S2) linking these conformations with B3LYP/6-31G­(d,p), we found an energy barrier of approximately 2 kcal/mol, upon correcting ZPE, along with an imaginary frequency of approximately 10i cm^–1^. Thus, our conformational analysis indicates that both conformers are likely to be thermally accessible in solvent and may coexist in equilibrium under experimental conditions. As is well known, small energy variations among these conformers are expected using different levels of theory.

As a complementary analysis, we have performed a QTAIM-based analysis^9^ to identify some bond critical points, i.e., [3, −1], associated with the intramolecular H-bond of the enol, which enables a good estimation of the interaction energy in each conformer. Using a linear relationship,^10^ we have obtained the ΔE*_H‑bond_ values of −10.9 and −11.7 kcal/mol for III and III′, respectively, by employing B3LYP/6-31G­(d,p), which are comparable results. A similar trend is found for the Br-substituted compound. These findings suggest that the Franck–Condon absorption due to the intramolecular H-bond formation in both conformers exhibits similar strengths, confirming that both III and III′ are possible candidates for undergoing ESIPT. To be more precise, an average calculation involving several uncorrelated conformers^5,6^ should be a more accurate method, although it is computationally more expensive.

Considering now the vertical excitation from S_0_ to S_1_ for the Br-substituted compound in both conformers III and III′, experimental data^3^ have reported an absorption maximum at 296.4 nm in *n*-hexane. Our TD-DFT calculations at the CAM-B3LYP/6-31G­(d,p) level yield wavelengths of 296.3 and 298.9 nm for conformers III and III′, respectively, showing excellent agreement with the experimental result. A similar trend is noted in 1,4-dioxane. The experimental absorption maximum is 295.6 nm, while our computed values are 296.7 and 299.0 nm for III and III′, respectively. It is also worth mentioning that the same orbitals are involved in the Franck–Condon transition from S_0_ to S_1_, as displayed in Figure 2. These findings confirm that both conformers absorb in the same spectral region, indicating that conformer III, despite not being the global minimum on the potential energy surface, remains a relevant conformer for the photochemical process.

**Figure 2:**
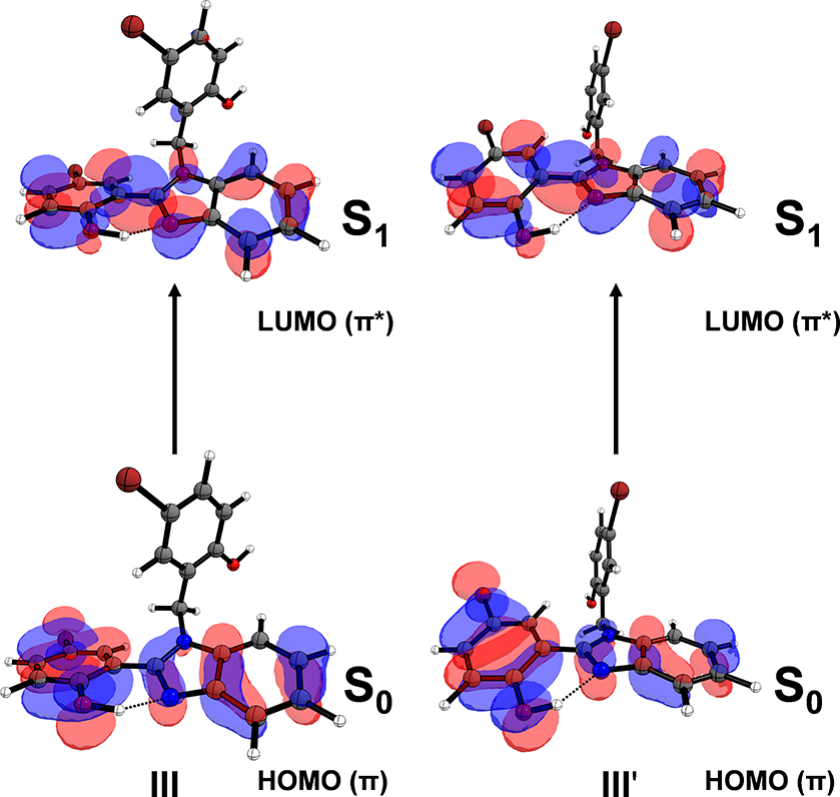
Illustration of the molecular orbitals involved in the absorption process in conformers III and III′.

Considering now the vibrational relaxation of conformer III in S_1_, it leads to keto conformer IV, still in S_1_, as previously discussed in ref 2. Considering the case of the vibrational relaxation of III′ in S_1_, the process results in the formation of IV′. Although the experimental results do not suggest the persistence of the keto form in the excited state, the energy difference ΔE_(IV–IV′)_ in S_1_ is only 1.2 kcal/mol. Taking into account the keto form in the ground state, we also identify a similar structure to that reported by Antonov et al.^1^ (Figure 3b). In fact, this structure corresponds to a possible conformer of V, described in ref 2. The optimized geometries at the B3LYP/6-31G­(d,p) level are displayed in Figure 3.

**Figure 3:**
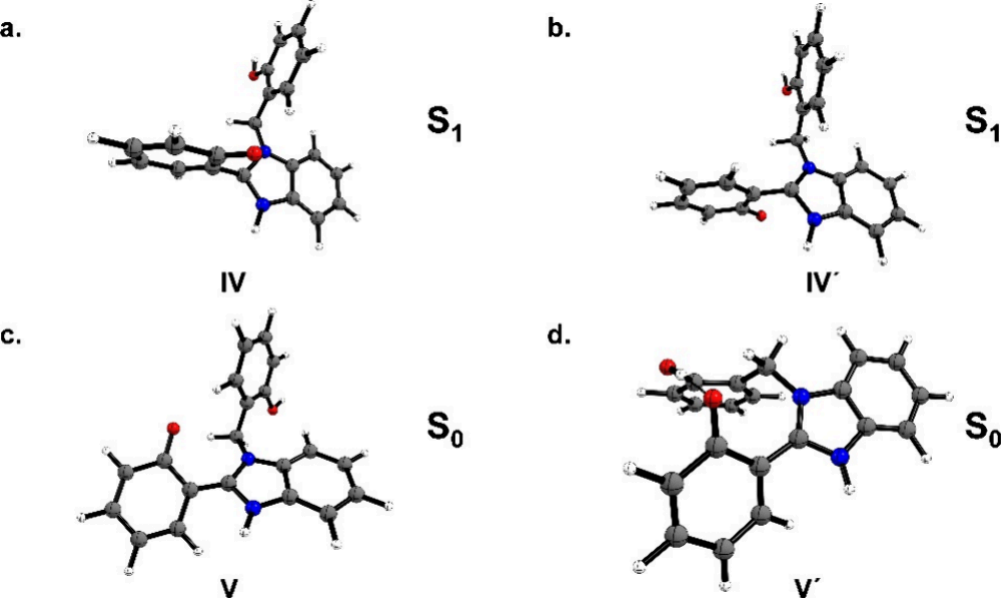
Optimized structures of the nonsubstituted compound with B3LYP/6-31G­(d,p) for conformer IV (a), conformer IV′ (b), conformer V (c), and conformer V′ (d), representing an alternative geometry for conformer V.

Finally, regarding the decay of these possible excited enolic forms to S_0_, the formation of the H-bond in V′ leads to a stabilization of –2.3 kcal/mol compared to V. Concerning the Stokes shift, the values associated with (III → V) and (III′ → V′) are 98.7 and 72.0 nm, respectively, which are still comparable values. Hence, the new conformers obtained by Antonov et al.^1^ contribute to an improved understanding of the ESIPT process in benzimidazole derivatives. Notwithstanding, considering the wide variety of the conformational degree of freedom of these compounds as well as the speed of ESIPT, these new results show that our previous contribution is still a possible picture for describing the benzimidazole derivatives in solution.

## Supplementary Material



## Notes

Conformational analysis (Figure S1 and Table S1), transition state (Figure S2), and Cartesian coordinates (PDF)
