# Supplementary material for: Reply to the Comment on “Influence of Solvents and Halogenation on ESIPT of Benzimidazole Derivatives for Designing Turn-on Fluorescence Probes”
Source: ACS Omega. 2025 May 27;10(22):22302–4. doi: 10.1021/acsomega.5c04406 (PMC12163653; doi:10.1021/acsomega.5c04406)
Supplement: Supplementary file 1 [file ao5c04406_si_001.pdf]

Supporting Information

Reply to the Comment on "Influence of solvents and halogenation on ESIPT of benzimidazole derivatives for designing turn-on fluorescence probes"

Murillo H. Queiroz[1], Joel. L. Nascimento[1], Tiago V. Alves[1], Roberto Rivelino[2,*], Sylvio Canuto[3]

[1]Departamento de Físico-Química, Instituto de Química, Universidade Federal da Bahia - Rua Barão de Jeremoabo, 147, 40170-115, Salvador, Bahia, Brazil

[2]Instituto de Física, Universidade Federal da Bahia, 40210-340 Salvador, Bahia, Brazil

[3]Instituto de Física, Universidade de São Paulo, CP 66318, 05315-970 São Paulo, SP, Brazil

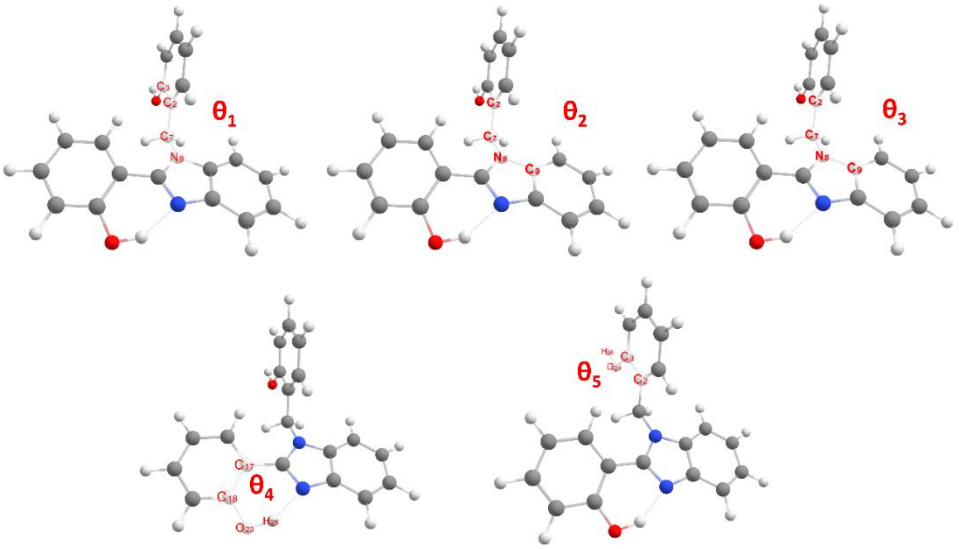

Figure S1. Relevant dihedral angles for the conformational analysis.

Table S1. Dihedral angles, rovibrational partition function at 298.15 K (Q_{vr}), relative contribution of each conformer to the total partition function (X), and Gibbs free energy (G). All calculated at B3LYP/6-31G(d,p).

Conformer	Angles (θ_1_θ_2_ θ_3_ θ_4_θ_5)	Q_{vr}	X	G (kcal/mol^{-1})
1	177_270_159_008_180	5.184E+13	0.95447	0.000
2	117_298_208_348_181	5.042E+13	0.02016	2.286
3	302_294_152_010_179	2.539E+13	0.00795	2.837
4	047_072_204_350_335	2.435E+13	0.00636	2.969
5	063_048_153_012_180	3.860E+13	0.00669	2.939
6	323_106_198_353_017	4.613E+13	0.00247	3.530
7	047_254_159_007_181	2.527E+13	0.00087	4.149
8	058_043_152_012_346	4.506E+13	0.00102	4.051
9	305_307_305_182_353	3.276E+13	0.00000	7.860
10	247_067_305_349_179	8.518E+13	0.00000	8.312
11	038_061_054_017_298	2.295E+13	0.00000	9.228
12	183_281_308_353_178	7.548E+13	0.00000	8.635
13	175_283_056_012_180	1.901E+14	0.00000	8.158
14	246_071_304_183_178	9.658E+13	0.00000	8.925
15	164_288_308_180_182	9.800E+13	0.00000	8.606
16	291_311_291_323_349	5.620E+13	0.00000	9.345
17	295_308_054_011_181	7.077E+13	0.00000	9.259
18	176_279_056_178_181	1.917E+14	0.00000	8.535
19	289_298_057_014_355	8.345E+13	0.00000	9.634
20	065_055_304_183_180	6.987E+13	0.00000	9.807
21	056_052_300_183_344	1.199E+14	0.00000	9.615
22	236_236_050_025_032	2.320E+13	0.00000	11.204
23	294_105_305_185_020	9.406E+13	0.00000	9.969
24	240_066_099_178_178	1.709E+14	0.00000	9.828
25	178_279_127_180_180	1.325E+14	0.00000	9.944
26	045_065_233_181_337	5.667E+13	0.00000	11.490
27	222_276_308_344_064	1.715E+13	0.00000	12.591
28	060_039_059_179_184	7.788E+13	0.00000	12.896

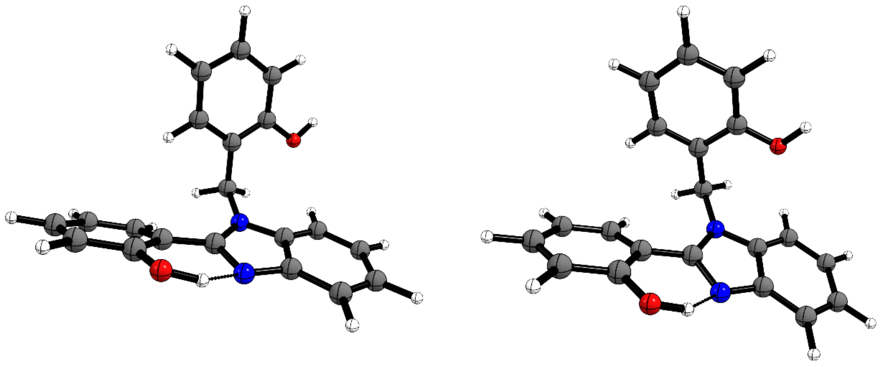

(a) (b)

Figure S2. (a) Guess and (b) optimized structures of a possible transition state (TS) linking conformers III and III'.

Cartesian coordinates of the guess and optimized structures of the TS at the B3LYP/6-31G(d,p) level of theory.

Guess structure				TS			
6	-2.773996000	1.455261000	-0.832883000	6	-2.877737000	1.626507000	-1.102050000
6	-1.419494000	1.438080000	-0.490638000	6	-1.559251000	1.532236000	-0.649170000
6	-0.845448000	2.434447000	0.329248000	6	-0.993806000	2.515772000	0.189646000
6	-1.638946000	3.450277000	0.880253000	6	-1.745703000	3.624089000	0.599915000
6	-2.993336000	3.446967000	0.569872000	6	-3.056826000	3.721027000	0.149262000
6	-3.546845000	2.479142000	-0.293437000	6	-3.612676000	2.735190000	-0.690682000
7	-0.380963000	0.570894000	-0.832489000	7	-0.552580000	0.591922000	-0.869367000
7	0.509874000	2.201271000	0.446556000	7	0.316987000	2.184984000	0.462421000
6	0.767042000	1.103002000	-0.257192000	6	0.566668000	1.052494000	-0.186544000
6	2.132934000	0.581141000	-0.358956000	6	1.903557000	0.450339000	-0.140822000
6	3.062936000	0.904391000	0.672714000	6	2.739823000	0.748251000	0.974313000
6	4.338962000	0.315213000	0.658664000	6	4.005563000	0.149905000	1.069772000
6	4.721704000	-0.520605000	-0.383518000	6	4.479703000	-0.672915000	0.056892000
6	3.855813000	-0.758623000	-1.457513000	6	3.702706000	-0.908195000	-1.083020000
6	2.583662000	-0.198952000	-1.440734000	6	2.433834000	-0.348586000	-1.169742000
8	2.768500000	1.756154000	1.672358000	8	2.373408000	1.591664000	1.956167000
1	1.898284000	2.187259000	1.444167000	1	1.553509000	2.063427000	1.644537000
1	4.162658000	-1.384566000	-2.291199000	1	4.086119000	-1.514064000	-1.897532000
6	-0.617521000	-0.776432000	-1.402027000	6	-0.797015000	-0.725841000	-1.487768000
6	-0.883038000	-1.887084000	-0.392537000	6	-0.761171000	-1.906330000	-0.535630000
6	-2.196667000	-2.304593000	-0.102487000	6	-1.765353000	-2.074840000	0.436277000
6	-2.433480000	-3.306372000	0.845313000	6	-1.749059000	-3.175734000	1.296635000
6	-1.363030000	-3.937516000	1.469771000	6	-0.738754000	-4.130644000	1.188541000
6	-0.050679000	-3.568324000	1.167432000	6	0.252450000	-3.993630000	0.218219000
6	0.168195000	-2.536894000	0.255832000	6	0.230242000	-2.886786000	-0.631698000
1	-1.464170000	-0.680522000	-2.084092000	1	-1.784812000	-0.653209000	-1.946451000
1	0.241940000	-1.053852000	-2.004169000	1	-0.089662000	-0.887396000	-2.301885000
1	0.781406000	-4.070907000	1.644345000	1	1.038442000	-4.735625000	0.124658000
8	-3.245629000	-1.697088000	-0.772187000	8	-2.751295000	-1.131599000	0.481814000
1	-4.065150000	-2.093926000	-0.490339000	1	-3.356942000	-1.327414000	1.209187000
1	-3.214032000	0.697554000	-1.468164000	1	-3.325687000	0.871368000	-1.738114000
1	-1.200837000	4.201763000	1.530396000	1	-1.304485000	4.378749000	1.242830000
1	-3.643384000	4.199346000	1.000444000	1	-3.665304000	4.570144000	0.445333000
1	-4.603489000	2.529491000	-0.541487000	1	-4.639840000	2.841298000	-1.026412000
1	5.021319000	0.546428000	1.471249000	1	4.606518000	0.376105000	1.944304000
1	5.708790000	-0.971141000	-0.373402000	1	5.468832000	-1.113395000	0.143058000
1	1.934945000	-0.345612000	-2.297268000	1	1.853887000	-0.506521000	-2.070774000
1	-3.471544000	-3.575421000	1.096957000	1	-2.533525000	-3.284125000	2.042128000
1	-1.561134000	-4.712712000	2.200118000	1	-0.734492000	-4.982308000	1.862141000
1	1.176437000	-2.226030000	0.039559000	1	1.006314000	-2.775403000	-1.381856000
